# Assessment of Acellular Dermal Matrix-Associated Complications With Silicone Implants

**DOI:** 10.7759/cureus.92664

**Published:** 2025-09-18

**Authors:** Eduardo De Faria Castro Fleury, Pryscilla Ferreira, Kamilly A Ferreira

**Affiliations:** 1 Radiology, Instituto Brasileiro de Controle do Câncer/Hospital São Camilo, São Paulo, BRA; 2 Breast Radiology, Instituto Brasileiro de Controle do Câncer/Hospital São Camilo, São Paulo, BRA

**Keywords:** acellular human dermal matrix, general surgery and breast cancer, mesh repair, plastic and reconstructive surgery, silicone breast prostheses

## Abstract

Acellular dermal matrix (ADM) is defined as an allograft composed of sterile, decellularized, regenerative dermal tissue matrix for soft tissue reconstructive and transplantation purposes. The epidermal layer is removed from the dermal layer to minimize immunological response in the ADM recipient. The idea of using ADM is to improve breast reconstruction and promote faster recovery with fewer complications.

It is expected that ADM will reduce capsular contracture, the most frequent complication of silicone implant surgeries. However, some complications have been related to ADM in breast reconstructive surgeries. In such cases, imaging scans, such as magnetic resonance imaging (MRI) and ultrasonography (USG), can aid in diagnosing and monitoring complications.

In this case series, we share our experience regarding ADM complications in our clinical practice. We showed the most common complications of ADM associated with breast implants in eight patients referred to USG, MRI, mammography, and computed tomography. The more frequent findings were capsular contracture, folded ADM, intracapsular collection, and pericapsular impairment. We also divided the findings into early and late complications.

ADM and its complications associated with breast implants can be diagnosed and followed up with breast imaging scans. Knowledge of ADM presentation and its complications is imperative for diagnostic purposes, managing, and following up patients who undergo these procedures.

## Introduction

The acellular dermal matrix (ADM) was first described by Brueing and Warren in 2005 in reconstructive breast surgery [1]. ADM is now commonly used in breast reconstructive surgery (including in breast cancer patients), post-lumpectomy filler, post-mastectomy (two-stage reconstruction), immediate breast reconstruction with implants, or correction surgeries of visible implant rippling and symmastia [2]. The main indication for ADM is to prevent capsular contracture, the most reported complication associated with silicone implants, and to improve implant stabilization.

There has been a surge in the use of ADM in reconstructive and aesthetic surgery; however, the usage in silicone implant surgery is controversial. Currently, the FDA does not recommend ADM for silicone implant surgeries. The idea of using ADM is to improve breast reconstruction and promote faster recovery. ADM is prepared by the process of native dermal tissue de-epithelialization (removing all antigenic material from the tissue to prevent rejection or inflammation) of human cadaveric donor skin (allogeneic) or mammalian skin donor sources (xenogeneic). Therefore, ADM can be from human (most commonly used), fetal bovine, or porcine origin [3,4]. ADM consists of intact collagen fibers and bundles, proteins, intact elastin, hyaluronic acid, fibronectin, fibrillar collagen, type VI collagen, vascular channels, and proteoglycans [1-4].

Currently, there is a lack of knowledge of the main ADM-related complications, the diagnosis, and follow-up strategies for these purposes [4,5]. In a 2021 safety communication, the FDA stated: "Be aware that the FDA has not approved or cleared any ADM products for use in implant-based breast reconstruction" [6].

This case series presents imaging findings, including mammography, ultrasonography (USG), computed tomography (CT), and magnetic resonance imaging (MRI), related to ADM complications in breast surgeries with silicone implants. The presentation is composed of: (a) expected findings, (b) early complications, and (c) late complications. We present imaging findings of eight patients with silicone implants and ADM and describe the main findings, such as capsular contracture, folded ADM, intracapsular collection, and pericapsular impairment.

## Case presentation

Imaging findings

**Figure 1:**
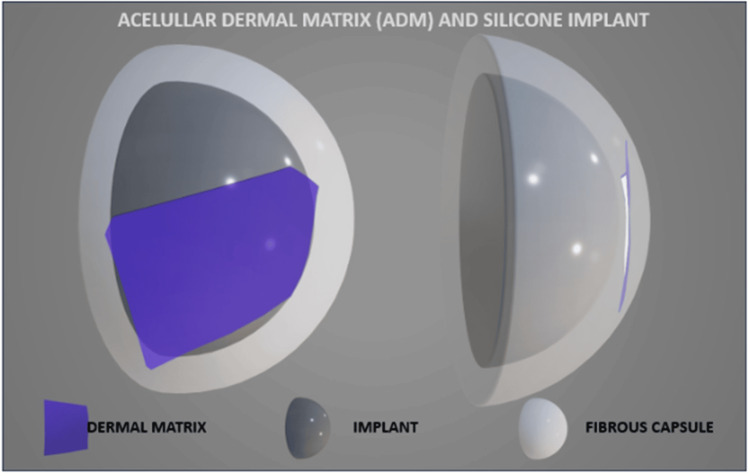
Acellular dermal matrix (ADM) and silicone implant Schematic illustration of the relationship between the fibrous capsule, ADM, and silicone implant. ADM is in a virtual space between the silicone implant and the fibrous capsule. Image credits: Eduardo de Faria Castro Fleury.

**Figure 2:**
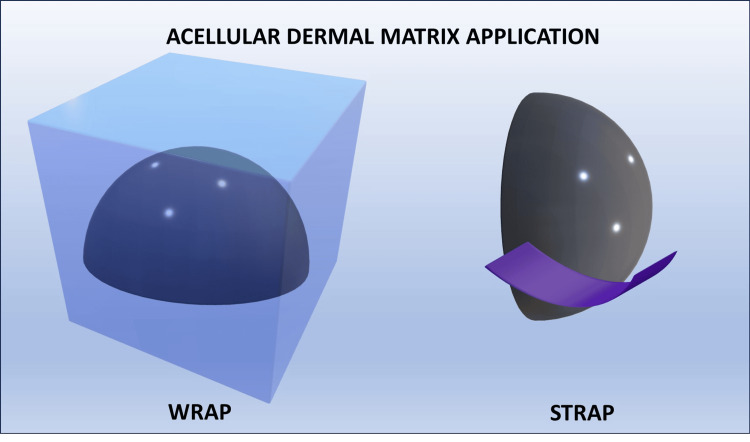
Acellular dermal matrix (ADM) insertion types: WRAP vs. STRAP Schematic illustration of the relationship between the fibrous capsule, ADM, and silicone implant. ADM is in a virtual space between the silicone implant and the fibrous capsule. Image credits: Eduardo de Faria Castro Fleury.

For pre-pectoral implants, ADM covered the entire implant (wrap). For retropectoral implants, ADM covered the lower outer part of the implant where it is not covered by the pectoralis major muscle (strap). ADM could cover only the anterior aspect of the implant in some cases (partial coverage). ADM was also used in pre-pectoral breast reconstruction in nipple-skin sparing mastectomies (Figures 1, 2) [2].

**Figure 3:**
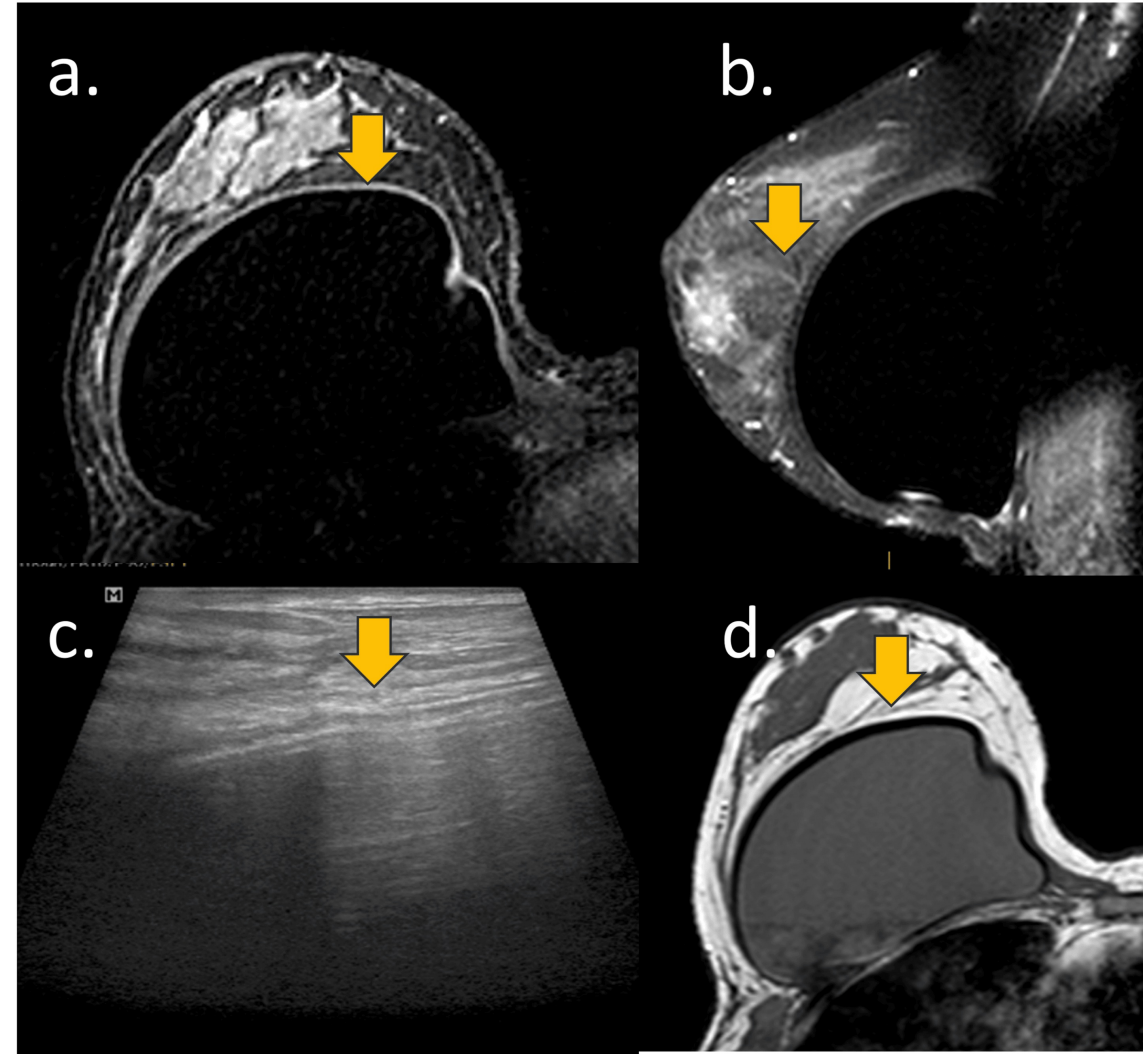
Expected acelullar dermal matrix (ADM) with silicone implants A 52-year-old woman with a retropectoral implant and ADM for five years. (a) Axial T1-weighted sequence, (b) sagittal STIR-weighted sequence, (c) breast USG, and (d) axial proton density-weighted sequence. (a, b, c, and d) The ADM is expected to integrate perfectly with the silicone implant and the fibrous capsule, remaining almost invisible to imaging methods, as shown by the yellow arrow. In these cases, the mammogram cannot be viewed. On MRI, pericapsular laminar tissue with marked hyposignal can be observed in all sequences. By studying ultrasound, the ADM can determine the attenuation of the sound beam.

In total coverage (wrap), ADM should evenly cover the entire implant; however, various degrees of folding can occur during surgery, and sutures could be placed at the edges of the implant to enhance stabilization. In partial coverage (strap) sutures, the implant could be secured between the ADM and the pectoralis muscle. After implantation, ADM could be vascularized and integrated into the surrounding tissue (Figure 3) [1,2].

If ADM was not fully integrated with the host's tissue, it might appear as a mass-like structure. ADM folding and suture sites might create novel mass-like structures in imaging files. The typical shape of ADM is either a sheet-like or an oval mass, especially when it is presented as a palpable mass [7]. The typical thickness of ADM is 1-1.5 mm and may increase in thickness after implantation in vivo over time. ADM integration into the fibrous capsule and pericapsular tissues may exhibit different enhancements on imaging studies, depending on its various stages of vascularization [2].

Early complications

Usually, early complications were associated with the acute inflammatory process. The primary patient's complaint of breast stiffness was edema and pain. Some patients might also present with enlargement of the breast. In acute episodes, an intracapsular collection was present. Early complications developed over the first two years after ADM placement [1-3].

**Figure 4:**
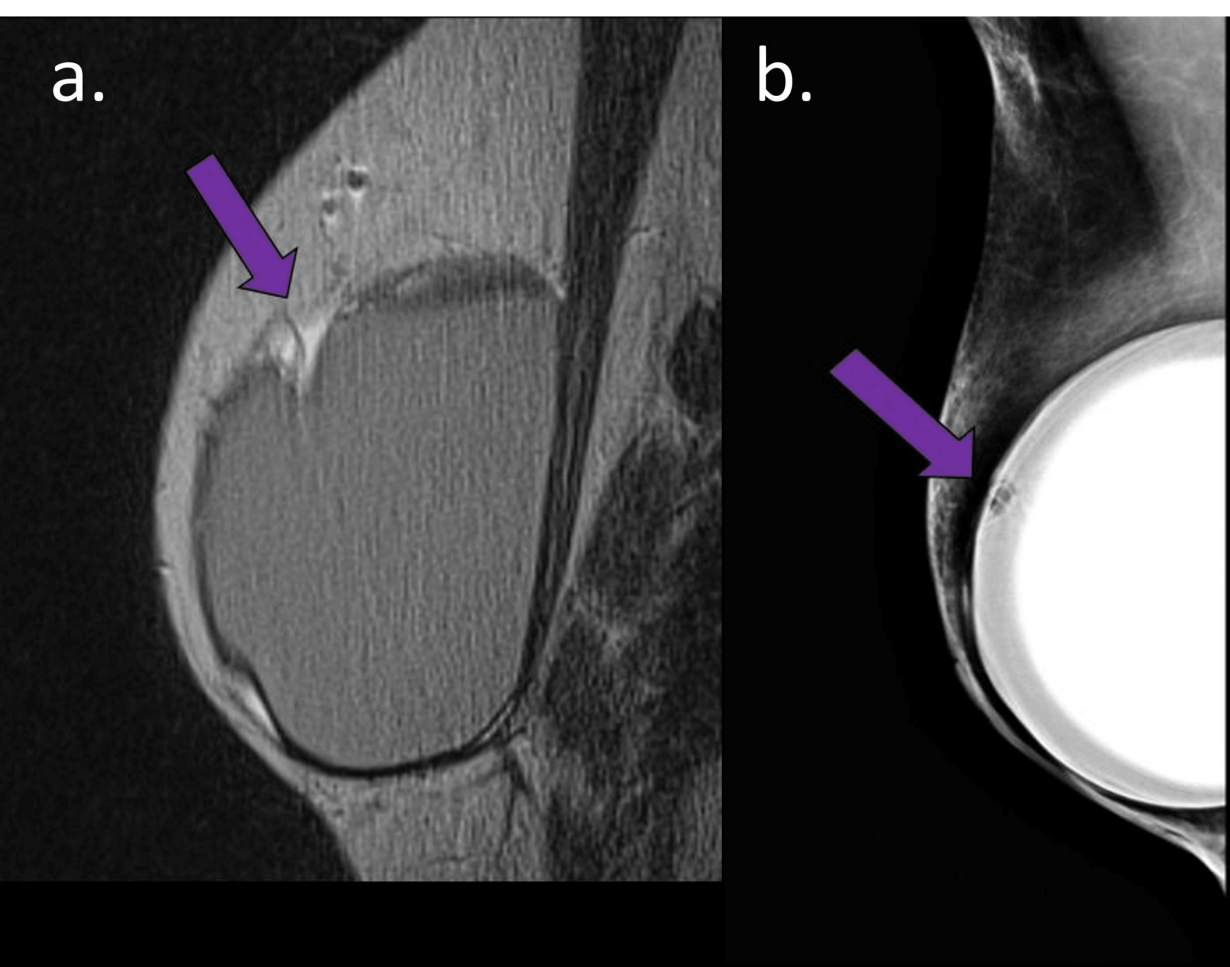
Mammography findings of acellular dermal matrix (ADM)-related complications A 60-year-old woman with a mastectomy and reconstruction with silicone implant and ADM for three years. (a) Sagittal DP-weighted sequence and (b) and breast mammography in oblique view. (a) Foldable intracapsular hyposignal mass (purple arrow) associated with intracapsular collection on MRI Mammogram: (a) In the same site as MRI findings and (b) radial fold with hypotransparency material inside (purple arrow)

Mammography showed radial folds, and signs of intracapsular collection were the main findings (Figure 4).

**Figure 5:**
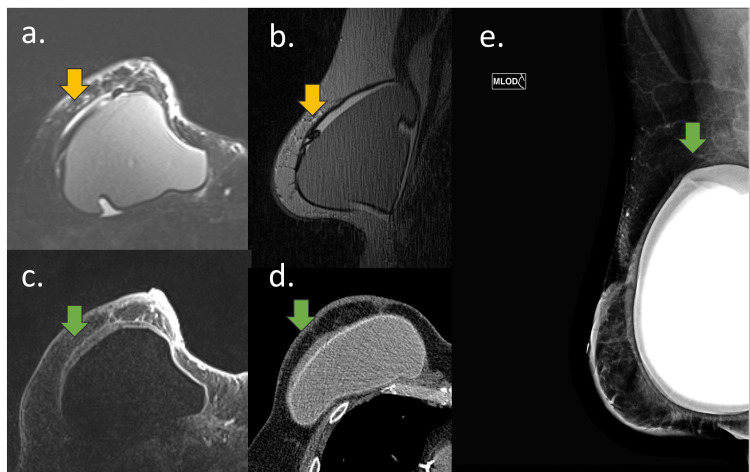
Early complications of acellular dermal matrix (ADM) A 55-year-old woman with an adenomastectomy reconstructed with a breast implant and ADM for one year. (a) Axial STIR-weighted sequence, (b) sagittal DP weighted sequence, (c) axial post-contrast sequence, (d) breast tomography, and (e) mammography oblique view MRI: (a, b, and c) Foldable intracapsular hyposignal tissue (yellow arrow) associated with intracapsular collection in green arrow. CT: (d) Intracapsular collection with heterogeneous material in green arrow and (e) intracapsular collection (green arrow) in mammography. The implant is rotated.

**Figure 6:**
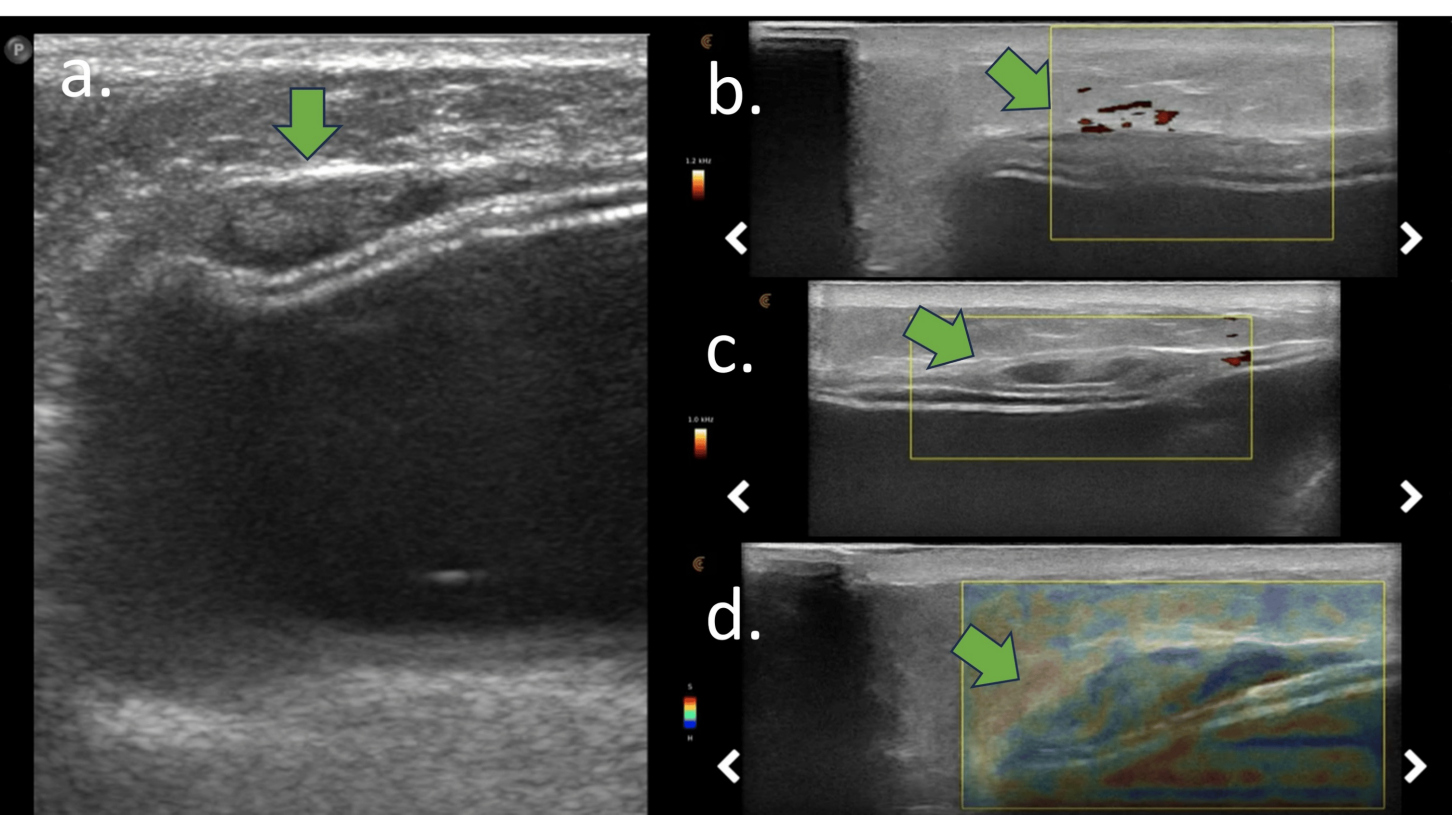
Ultrasound findings of early acellular dermal matrix (ADM) complications USG of the same patient as in Figure 5. (a) B-mode, (b) power-Doppler mode, (c) power-Doppler mode, and (d) elastography. (a, b, c, and d) Intracapsular heterogeneous tissue (green arrow) associated with intracapsular collection and fibrous capsule thickening. (b and c) Increased vascularization in the fibrous capsule. (d) The intracapsular tissue has moderate to high stiffness in elastography.

USG showed an intracapsular mass that attenuates the ultrasound beam and could be associated with intracapsular collections. Fibrous capsule thickening with increased pericapsular and intracapsular vascularization might also be observed. The Doppler scans revealed increased vascularization during the acute process. Elastography might be a complementary tool for confirming tissue stiffness. Another ultrasound finding associated with ADM is irregularity of the silicone implant surface, accompanied by snowstorm artifacts in its periphery (Figures 5, 6).

**Figure 7:**
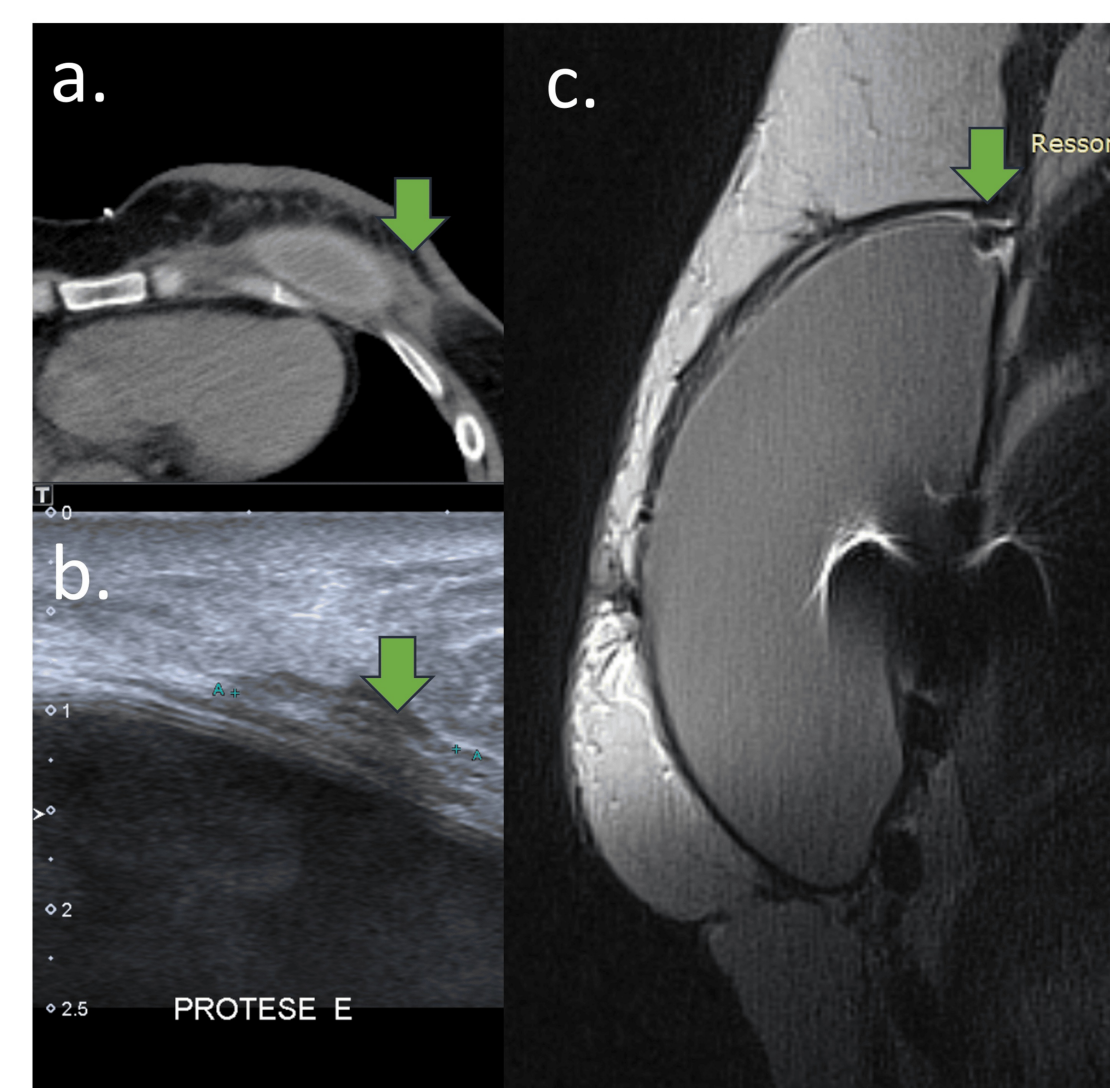
Early complications of acellular dermal matrix (ADM) on MRI A 61-year-old woman with a retropectoral implant and ADM. (a) CT, (b) B-mode USG, and (c) sagittal PD-weighted sequence CT: (a) Intracapsular collection with heterogeneous material and skin thickening (green arrow). USG: (b) Heterogeneous pericapsular tissue with fibrous capsule and implant surface irregularity. MRI: (c) Foldable intracapsular tissue

CT might show an intracapsular collection with heterogeneous tissue and pericapsular edema (Figure 7).

**Figure 8:**
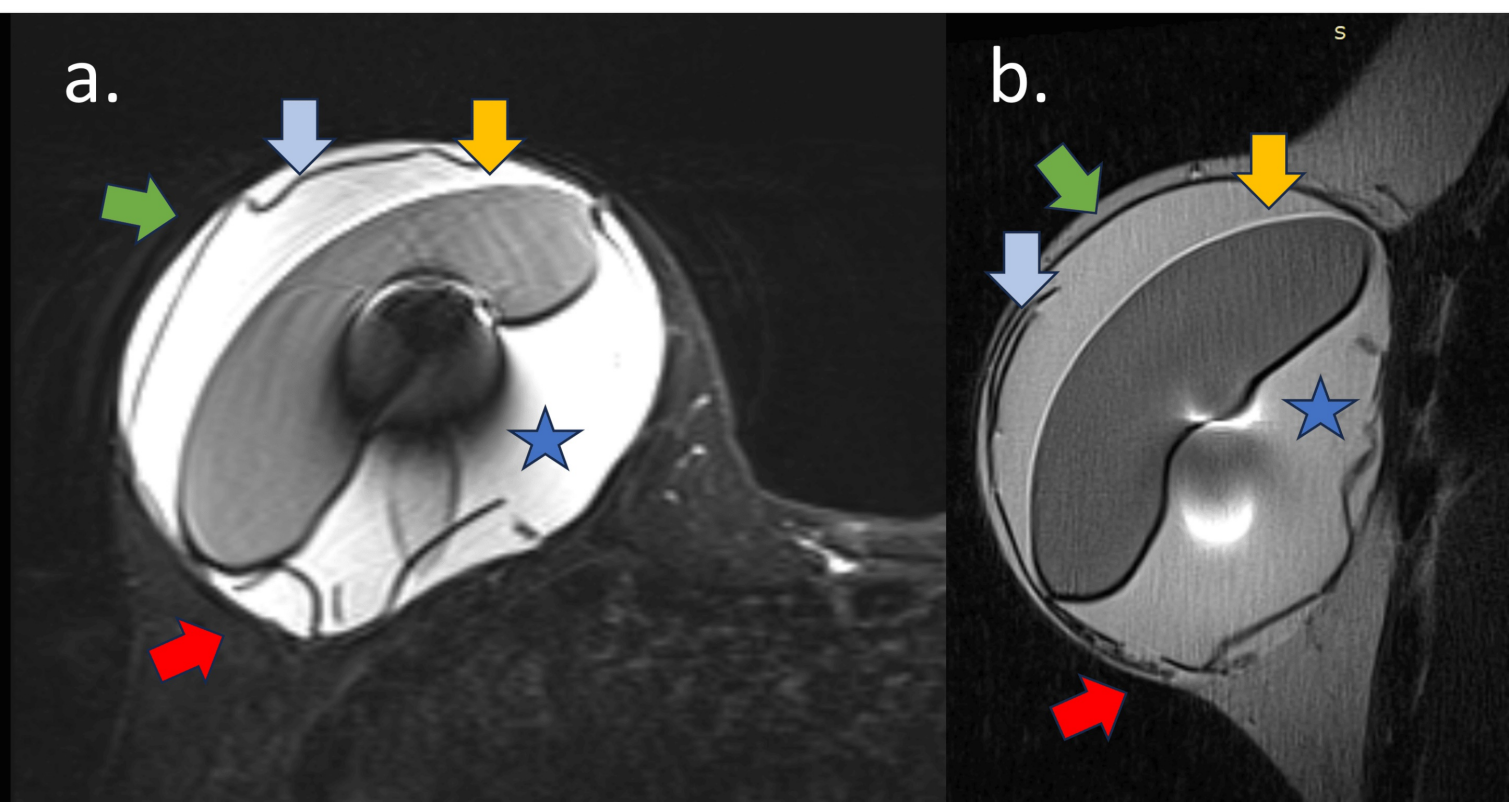
Early complications of ADM on MRI A 44-year-old woman with a retroglandular implant and ADM for six months. (a) Axial STIR-weighted sequence and (b) sagittal PD-weighted sequence. Voluminous intracapsular collection (blue star) with fibrous capsule irregularity (green arrow), implant surface (yellow arrow), intracapsular heterogeneous tissue compatible with ADM (blue arrow), and intracapsular vegetation (red arrow)

**Figure 9:**
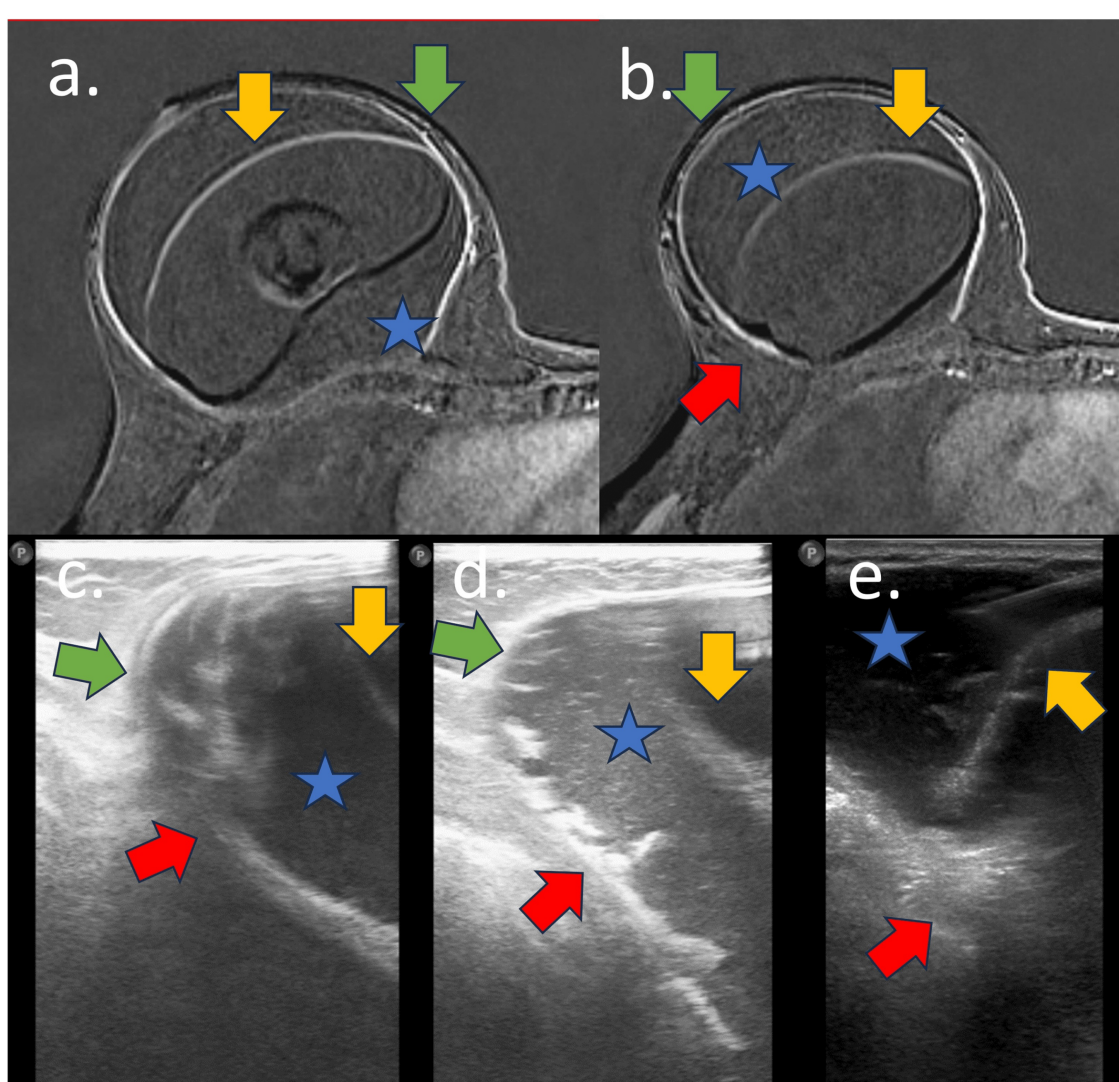
Early complications of acellular dermal matrix (ADM): USG and MRI Same patient as Figure 8. (a and b) Axial post-contrast sequence and (c, d, and e) B-mode USG. Voluminous intracapsular collection (blue star) with fibrous capsule irregularity (green arrow), implant surface (yellow arrow), intracapsular heterogeneous tissue compatible with ADM (blue arrow), and intracapsular vegetation (red arrow)

**Figure 10:**
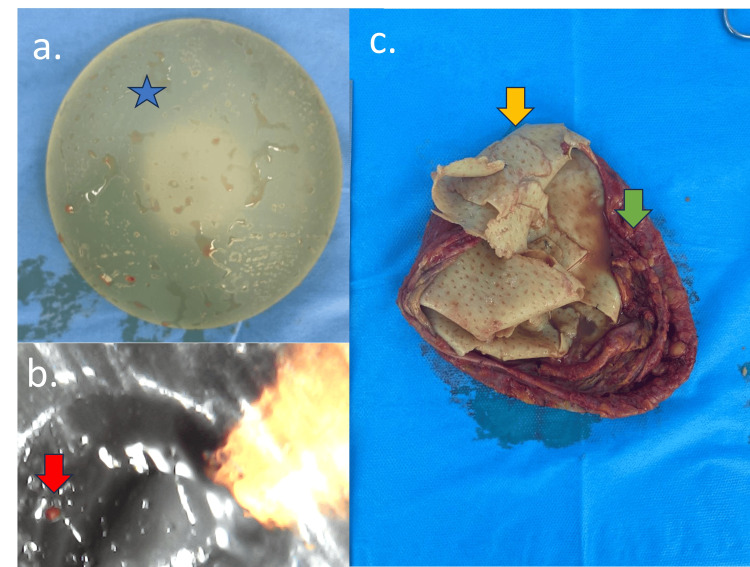
Early complication of acelullar dermal matrix (ADM): surgical specimen Same patient as Figures 8, 9. (a) Implant macroscopy, (b) microscopy, and (c) surgical specimen of the fibrous capsular and ADM. Silicone implant (blue star) with fibrous capsule irregularity (green arrow), implant surface irregularity (red arrow), and ADM (yellow arrow)

MRI showed a marked intracapsular hypointense signal material in all sequences, which was sometimes observed in a folded appearance, that could be associated with capsular contracture, pericapsular edema, intracapsular collection, and implant surface enhancement (Figures 8-10) [2]. When folded, ADM might appear as an intracapsular mass with late contrast enhancement and could be associated with intracapsular seroma and pericapsular edema. Most often, silicone implant complications were present in these cases, such as silicone-induced granuloma of the breast implant capsule, water-droplet signals, and enhancement of the silicone surface.

Late complications

**Figure 11:**
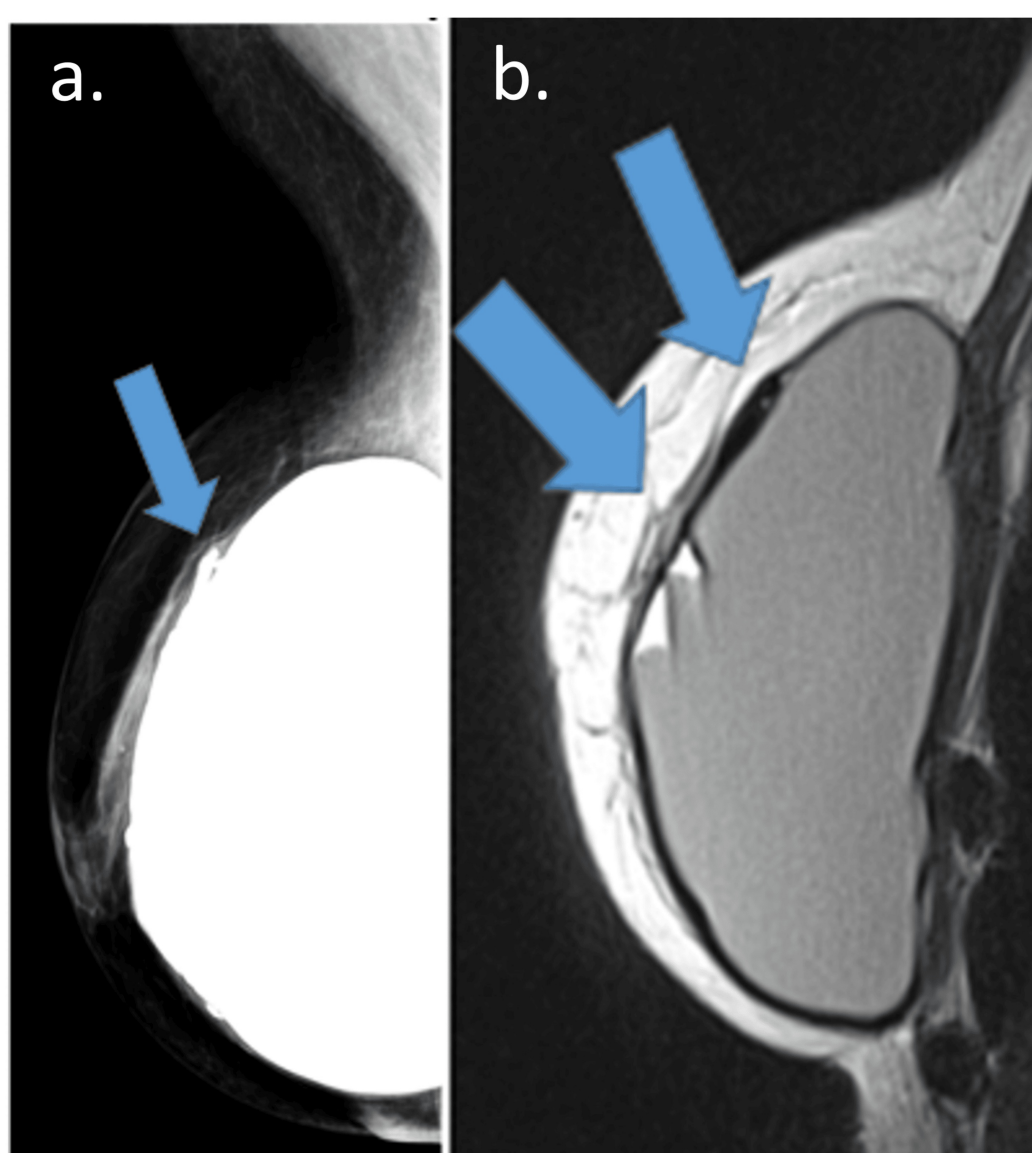
Late complication of acelullar dermal matrix A 42-year-old woman with an adenomastectomy for 10 years. (a) Mammography oblique view and (b) sagittal PD sequence image (a and b) Gross calcification in the pericapsular space (blue arrow) on mammography and MRI

The late complications of ADM were related to granulomatous tissue, which involved the ADM and resulted in gross, benign calcifications (Figure 11).

## Discussion

The silicone implant is an adjuvant that degrades the surface and promotes gel bleeding over time. As the human body creates a fibrous capsule to protect the foreign body, exposure of the fibrous capsule to this free silicone may induce an inflammatory process, resulting in capsular contracture. ADM is believed to be inert to the human body, biodegradable, and biocompatible. The application of ADM in silicone implant surgeries aims to reduce the incidence of silicone implant-related complications [1-3].

The main complications observed in our clinical practice are intracapsular seroma, capsular contracture, abscess, hematoma, fat necrosis, and calcification. As a novel technique, there is a lack of knowledge regarding complications and protocols for follow-up of these patients, as well as a specific lexicon to describe the imaging findings.

In the acute phase, USG and MRI can provide information regarding the ADM status and related complications. Notably, intracapsular collections, fibrous capsule thickening and enhancement, and pericapsular edema are the most significant imaging findings.

In 2022, Kim et al. [7] proposed an ultrasonographic classification for ADM complications, which includes type 1 (focal thickening with decreased echogenicity), type 2 (diffusely hyperechoic), and type 3 (bright echogenic spot). The study shows abnormal findings in 47.8% of the 207 ultrasounds performed. However, the study did not consider the influence of breast implants on complications and did not compare the results with MRI, the gold standard for ADM-related complications.

Many of the complications observed after ADM are similar to those related to silicone implants, which are generally associated with gel bleeding from the breast implant. The implant-related complications are capsular contracture, intracapsular collection, edema associated with phlogistic signs, breast induration, among others [8]. However, in cases of breast reconstruction with a dermal matrix and silicone implant, it would be unwise to attribute the presented complications solely to the dermal matrix. However, it can be suggested that the combination of ADM with the prosthesis predisposes to complications. One factor that supports this hypothesis is the surgical suture fixation of the ADM to the adjacent tissue in the retropectoral implant, which alters the permeability of the implant.

Imaging in the postoperative condition serves to identify or rule out complications in symptomatic patients, such as pain, inflammatory conditions, or a new palpable finding. However, ADM makes diagnosis challenging due to its variable appearance and lack of knowledge of these complications. Mammography, due to the biplanar acquisition, provided less information for ADM evaluation. USG had good accuracy in diagnosing acute complications of ADM. CT was not suitable for evaluating ADM complications. Finally, MRI was the gold-standard imaging method for diagnosing ADM complications due to its multiplanar images and dedicated sequences for evaluating silicone implants and breast tissue.

**Figure 12:**
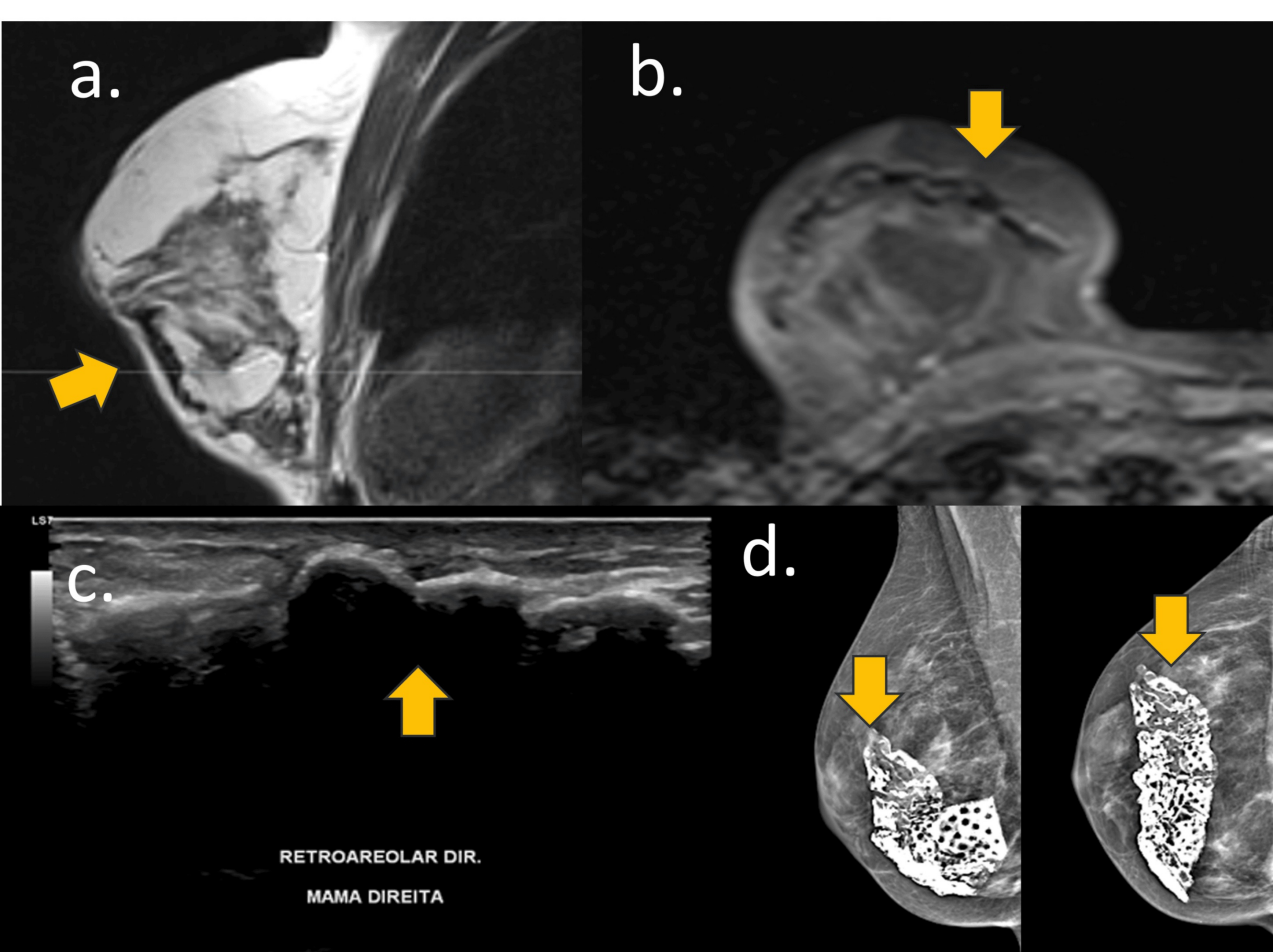
Marlex mesh A 67-year-old woman with a retropectoral implant and Marlex mesh for 30 years. (a) Sagittal DP-weighted sequence, (b) axial T1-weighted sequence, (c) breast USG, and (d) mammography An example of Marlex mesh during mastopexy 30 years ago. (a and b) Marlex mesh appears as a hypointensity signal on MRI, (c) hyperechoic mass with acoustic shadow on USG, and (d) calcified foreign body on mammography.

The use of synthetic mesh is not new in breast reconstruction surgeries. In the 1990s, Marlex Mesh was used for mastopexies, but its use was discouraged due to reported complications, mainly because it hindered cancer screening. The Marlex Mesh is indicated for chest wall reconstruction (Figure 12) [9].

We must be aware that the ADM application elicits a different biological response when inserted into the human body, especially when associated with breast implants. Hence, the applicability of the ADM in breast reconstructions remains debatable. There are still a few reports regarding the potential complications of ADM. In this pictorial essay, we share our experience to contribute to the assessment and interpretation of imaging findings, which will benefit patients by providing the most appropriate diagnosis, treatment, and follow-up.

## Conclusions

ADM and its complications associated with breast implants can be diagnosed and followed up with breast imaging scans. Knowledge of ADM presentation and its complications is imperative for diagnostic purposes, managing, and following up patients who undergo these procedures.
